# Peritonitis – the Western experience

**DOI:** 10.1186/1749-7922-1-25

**Published:** 2006-09-05

**Authors:** Mark A Malangoni, Tazo Inui

**Affiliations:** 1 Department of Surgery, Case Western Reserve University School of Medicine, MetroHealth Medical Center, Cleveland, Ohio, USA

## Abstract

Peritonitis is a common surgical emergency. This manuscript will provide an overview of recent developments in the management of peritonitis in the Western world. Emphasis is placed on the emergence of new treatments and their impact of outcomes.

## Background

Peritonitis can be defined in a variety of ways. Primary peritonitis is an infection of the peritoneal cavity usually occurring in patients with preexisting ascites that is not related to diseases of the abdominal or retroperitoneal viscera. Secondary peritonitis, the most common form of peritonitis, can occur due to spontaneous perforation of the gastrointestinal tract, intestinal ischemia, or following an operation. Tertiary peritonitis is a recurrent infection of the peritoneal cavity that follows an episode of either primary or secondary peritonitis [1,2]. Peritonitis can also be classified as diffuse or localized. Over time, peritoneal infection can coalesce to form an intraabdominal abscess. These two forms of peritonitis are often referred to collectively as intraabdominal infection.

There are a variety of factors influencing the reduction in mortality from peritonitis over the last century. Safer anesthetic techniques, improved understanding of perioperative fluid management, the advent of blood banking, improvements in critical care, more rapid and accurate diagnostic studies, and more effective antibiotics are some of the factors that have led to a reduction in mortality from peritonitis. On the other hand, a variety of challenges have arisen that threaten to offset these advances. Patients with intraabdominal infection are older, more commonly have comorbid diseases, often have associated immune suppression due to chronic diseases or their treatment, and more frequently have decreased physiologic reserve with either sub-clinical or evident organ failure. Although the bacterial flora of the gastrointestinal tract has remained relatively consistent over time, the widespread presence of antimicrobial resistance among patients with nosocomial as well as community-acquired infections has presented another challenge. This is particularly true for patients who have received previous antimicrobial treatment, inappropriate therapy, or have developed tertiary peritonitis where the pathogens are commonly resistant to front- line agents [2-5].

Much of what has been learned about the management of peritonitis has come from prospective randomized clinical trials. A number of important concepts have developed from these studies. We recognize that patients with appendicular sources of peritonitis have a lower mortality and improved outcomes compared to patients with non-appendicular sources [6]. Pancreatic necrosis incites a unique systemic inflammatory response, which is commonly associated with respiratory failure as well as other organ failures. Although pancreatic necrosis was once treated commonly with operation, non-operative management of sterile necrosis has become the norm due to the use of long-term prophylactic antibiotics to prevent infection and recognition than uninfected necrosis will usually resolve over time [7]. Pancreatic necrosis is thus excluded from most reviews of peritonitis as it will be from the present review.

The successful management of intraabdominal infection is predicated on the use of appropriate operative measures to address peritoneal infection. Prospective clinical trials have also taught us the importance of the concept of "source control" [2]. Source control encompasses all of the measures that eradicate the focus of infection, prevent continuing contamination, and restore functional anatomic relationships. This generally involves: 1) drainage of abscesses or infected fluid collections; 2) débridement of necrotic or infected tissues; and 3) definitive measures to control the source of contamination and to restore anatomy and function.

The response to intraabdominal infection depends upon the complex interaction of a variety of factors. The degree of microbial contamination, the site of origin of contamination and whether contamination is localized or diffuse are important. Previous operations or diseases can result in adhesions that may help localize infections. The systemic response to infection depends upon immune status as well as innate genetically coded responses to infection.

The diagnosis of intraabdominal infection is usually based on history and physical examination. Many patients will have abdominal computed tomography (CT) scanning to establish the diagnosis. CT is also useful to identify patients with localized abscesses who are candidates for percutaneous drainage rather than operation.

The treatment of intraabdominal infections is based on the restoration of normal homeostasis. Treatment principles include: 1) restoration of fluid and electrolyte imbalances; 2) administration of appropriate empiric antimicrobial therapy; 3) control of the source of infection; and 4) physiologic support of organ systems. Failure to address any of these important areas can lead to increased mortality, an increased incidence of organ failure, and prolonged hospital stay.

This article will review the recent advances in the treatment of intraabdominal infections in the Western world. Emphasis will be on the emergence of newer developments upon the outcome of treatment.

## Specific disease conditions

### Appendicitis

Acute appendicitis is the most common cause of intraabdominal infection in Western countries. It is associated with a lower mortality, shorter duration of hospital stay, and lower morbidity than other intraabdominal infections [6]. The advent of minimally invasive surgery has affected the treatment of acute appendicitis as laparoscopic appendectomy is being used increasingly to treat this disorder [8]. The results of laparoscopic appendectomy are better than open operation, particularly when patients have gangrenous appendicitis or early perforation with localized peritonitis and before an intraabdominal abscess or diffuse peritonitis has occurred. In this situation, laparoscopic appendectomy can be performed with a low conversion rate to open operation and acceptable results. Laparoscopic appendectomy is not recommended for patients who have a diffuse peritonitis as it is often more difficult to cleanse the peritoneal cavity of debris and infected fluid in this circumstance [9]. In general, complicated appendicitis is successfully treated with appendectomy and antibiotic management in greater than 90% of cases. The mortality in patients with this disorder is generally 1% or less.

Recent reports have demonstrated that antibiotics alone are useful to treat patients with early, non perforated appendicitis [10]. Non-operative management results in a recurrence rate of approximately 15%. Patients who present with perforated appendicitis and a localized right lower quadrant abscess can be treated successfully with percutaneous abscess drainage and antibiotics. Interval appendectomy is recommended because of an associated recurrence rate of 10–15%.

### Colon

Colonic perforations are the second most common cause for secondary peritonitis in the Western world, and colonic diverticulitis is the most common disease process resulting in perforation. Perforated colon cancer, ischemic colitis, and foreign body perforations also can lead to intraabdominal infection.

There has been an evolution in the management of colon perforation, particularly among patients with perforated diverticulitis. The three-stage operative approach involving abscess drainage with diverting colostomy followed by resection of the involved bowel with anastomosis and later closure of the protecting colostomy has been demonstrated to be inferior to a two-stage approach [11]. In the two-stage approach (Hartmann procedure), the abscess is drained and involved colon resected with formation of an end colostomy at the initial operation. The colostomy is closed and a definitive anastomosis is performed to the rectal stump at a later time.

More recently, a number of reports have suggested that primary resection and anastomosis is the preferred approach, even in the presence of diffuse peritonitis [12-15]. Resection with primary anastomosis is generally reserved for patients with less severe disease, have early contamination rather than advanced peritonitis, and who are in better physiologic condition as determined by performance status. Colonic obstruction has been identified as a risk factor for the development of post-operative complications after primary resection with anastomosis [15]. An alternative approach for patients who present with an acute diverticular abscess is percutaneous abscess drainage followed by single stage resection of the involved colon with primary anastomosis once the acute infection has resolved [16].

### Gastroduodenal

Gastroduodenal perforations have decreased significantly in Western countries due to the widespread adoption of medical therapies for peptic ulcer disease as well as the use of appropriate stress ulcer prophylaxis among critically ill patients. Operative management has migrated to the increased use of primary closure and non-resective techniques for the management of benign perforations as large as 3 cm in diameter [17]. The use of post-operative antibiotic treatment for associated *Helicobacter pylori *infection as well as proton pump inhibitors has increased the success of these management techniques. Resective approaches are usually reserved for patients with perforations due to gastric cancer.

### Small intestine

Jejunoileal perforations are relatively uncommon as a source of peritonitis in the Western world in contrast to Eastern countries [18]. Most small intestinal perforations are due to unrecognized traumatic injuries or intestinal ischemia. Treatment is most commonly resection of the involved segment with primary anastomosis. Some patients with intestinal ischemia may benefit from repeated laparotomy to assess the viability of marginally ischemic intestine as well as anastomotic integrity. An alternative to primary anastomosis in this circumstance is the use of resection with stapling of the remaining portions of the intestine. In this situation, primary anastomosis can be performed safely at the time of reoperation 24 to 48 hours later [19].

### Postoperative infections

Infections following elective operations on the gastrointestinal tract or the other abdominal viscera account for 20–25% of patients with peritonitis [6]. Abdominal CT scanning has been widely used to diagnose post-operative infections of the peritoneal cavity. These patients frequently present with localized infections that are amenable to percutaneous drainage. When operation is required, the principles of operation outlined above apply.

## Management of localized peritonitis

Patients with a localized intraabdominal abscess are often candidates for percutaneous drainage. This is usually done under CT or ultrasound guidance. Percutaneous drainage is most successful for patients with single abscesses that are accessible by a safe route. Patients with multiple abscesses, complex or multilocular abscesses, associated necrotic tissue, or who require resection of a neoplasm are usually better candidates for open drainage [20].

Both percutaneous and open drainage of intraabdominal abscesses have a similar rate of success. There is no doubt that percutaneous drainage is associated with less morbidity and a shorter length of stay. Mortality appears to be similar for these two techniques.

## Measures of successful treatment

Adequate source control can be achieved at initial operation in 90% or greater of patients. The need for reoperation in this group is less than 10%. When source control is not possible at the initial operation, the rate of reoperation is 30% or greater [21,22].

There is both a significant increase in mortality and worse long-term survival among patients with peritonitis who undergo planned relaparotomy compared to those who have relaparotomy on demand [23]. Exceptions include patients with intestinal ischemia, advanced tertiary peritonitis, infected ascites, or those who need to have reestablishment of intestinal continuity at a second operation.

## Role of antimicrobial therapy

The recommended antimicrobial regimens for patients with intraabdominal infections have been outlined by the Surgical Infection Society based on prospective randomized clinical trials (Table 1) [24,25]. Since this publication, additional antimicrobial regimens have been found to be of similar efficacy to these previously endorsed drugs [26,27]. Importantly, all of the recommended regimens are effective against gram negative enteric aerobic and anaerobic microorganisms. A recent review of prospective randomized studies of antibiotic regimens for secondary peritonitis of gastrointestinal origin in adults from the Cochrane Colorectal Cancer Group concluded that 16 antibiotic regimens had similar rates of clinical success [28]. There was no difference in mortality between any of these regimens.

**Table 1:** Recommended antimicrobial regimens for patients with intra-abdominal infection

**Single agents**
Ampicillin/sulbactam
Cefotetan
Cefoxitin
Ertapenem
Imipenem/cilastatin
Meropenem
Moxifloxacin
Piperacillin/tazobactam
Ticarcillin/clavulanic acid
**Combination regimens**
Aminoglycoside plus an antianaerobe agent (clindamycin or metronidazole)
Aztreonam plus clindamycin
Cefuroxime plus metronidazole
Ciprofloxacin plus metronidazole
Third-or fourth-generation cephalosporin (cefepime, cefotaxime, ceftazidime, ceftizoxime, or ceftriaxone) plus an antianaerobe

The use of appropriate empiric antimicrobial treatment has been associated with improved survival in a variety of clinical settings [5,29]. A recent study by Baré et al. has demonstrated that selection of an appropriate treatment regimen as recommended by the Surgical Infection Society was associated with a significant and marked improvement in successful treatment [5]. Mortality was not significantly reduced by the use of an appropriate regimen. This study was conducted retrospectively and only patients with community-acquired intraabdominal infections were included. These authors also identified colonic sites of infection, age ≥ 75 years, and a Charlson Index of one or greater as other factors associated with successful treatment.

It has been recognized for some time that patients who have intraabdominal infections and are treated with empiric antimicrobial therapy have a greater rate of treatment failure when resistant organisms are cultured [30,31]. The influence of *Candida *cultured from the peritoneal fluid has been  controversial, since this organism is not routinely treated by most empiric therapy regimens. Montravers and coworkers have demonstrated that the isolation of *Candida *from peritoneal cultures of patients with nosocomial peritonitis appears to be an independent risk factor for mortality [32]. In contrast, patients with community-acquired infections who have growth of *Candida *on culture were not at greater risk for death.

There has not been a consensus about the appropriate duration of treatment for intraabdominal infections. Some believe that antibiotics can be stopped once fever and leukocytosis have resolved, and gastrointestinal function has returned [25], while others recommend a specific duration of therapy [24]. The development of effective oral antimicrobials for the treatment of intraabdominal infections has led to a number of prospective randomized trials that have advocated switching to oral antibiotics once patients can tolerate a diet. [24,25,27] This has been advocated as a cost saving measure without clear data defining the duration of treatment. Taylor and colleagues have demonstrated that the use of postoperative oral antibiotics once intravenous antibiotics were stopped did not improve outcomes in patients with complicated appendicitis [33]. This study questions whether continued antibiotics are needed in patients once gastrointestinal function has returned.

## Conclusion

The clinical outcomes associated with secondary peritonitis are highly dependent upon the site of contamination (appendicitis vs others), as well as local and systemic factors. Recent developments in care have influenced the route and choice of operation. Improvements in antimicrobial therapy and results of prospective randomized clinical trials have identified a variety of effective antibiotics for the management of these disorders. There continues to be controversy about the optimal duration of antimicrobial therapy for secondary peritonitis.

## Competing interests

Dr. Malangoni has served as a consultant and received research funding from Astra-Zeneca, Bayer, Eli Lilly, Merck, Ortho-McNeill and Wyeth-Ayerst. Mr. Inui has no competing interests.

## Authors' contributions

MM contributed to the conception and design, acquisition of data, analysis and interpretation of data, and final approval of the manuscript.

TI was involved in acquisition of data and drafting of the manuscript.

The authors have read and approved the final manuscript.

## References

[B1] Malangoni MA (2003). Current concepts in peritonitis. Curr Gastroenterol Rep.

[B2] Marshall JC (2004). Intra-abdominal infections. Microbes Infect.

[B3] Malangoni MA (2000). Evaluation and management of tertiary peritonitis. Am Surg.

[B4] Nathens AB, Rotstein OD, Marshall JC (1998). Tertiary peritonitis: clinical features of a complex nosocomial infection. World J Surg.

[B5] Bare M, Castells X, Garcia A, Comas M, Egea MJ (2006). Importance of appropriateness of empiric antibiotic therapy on clinical outcomes in intra-abdominal infections. Int J Technol Assess Health Care.

[B6] Merlino JI, Malangoni MA, Smith CM, Lange RL (2001). Prospective randomized trials affect the outcomes of intraabdominal infection. Ann Surg.

[B7] Malangoni MA, Martin AS (2005). Outcome of severe pancreatitis. Am J Surg.

[B8] Nguyen NT, Zainabadi K, Mavandadi S, Paya M, Stevens CM, Root J, Wilson SE (2004). Trends in utilization and outcomes of laparoscopic versus open appendectomy. Am J Surg.

[B9] Liu S, Siewert B, Raptopoulos V (2002). Factors associated with conversion to laparotomy in patients undergoing laparoscopic appendectomy. J Am Coll Surg.

[B10] Styrud J, Eriksson S, Nilsson I, Ahlberg G, Haapaniemi S, Neovius G, Rex L, Badume I, Granstrom L (2006). Appendectomy versus antibiotic treatment in acute appendicitis. A prospective multicenter randomized controlled trial. World J Surg.

[B11] Howe HJ, Casali RE, Westbrook KC, Thompson BW (1979). Acute perforations of the sigmoid colon secondary to diverticulitis. Am J Surg.

[B12] Salem L, Flum DR (2004). Primary anastomosis or Hartmann's procedure for patients with diverticular peritonitis? A systematic review. Dis Colon Rectum.

[B13] Chandra V, Nelson H, Larson DR, Harrington JR (2004). Impact of primary resection on the outcome of patients with perforated diverticulitis. Arch Surg.

[B14] Zeitoun G, Laurent A, Rouffet F, Hay J, Fingerhut A, Paquet J, Peillon C, the French Association for Surgical Research (2000). Multicentre, randomized clinical trial of primary *versus *secondary sigmoid resection in generalized peritonitis complicating sigmoid diverticulitis. Br J Surg.

[B15] Gooszen AW, Tollenaar RA, Geelkerken RH, Smeets HJ, Bemelman WA, Van Schaardenburgh P, Gooszen HG (2001). Prospective study of primary anastomosis following sigmoid resection for suspected acute complicated diverticular disease. Br J Surg.

[B16] Stabile BE, Puccio E, vanSonnenberg E, Neff CC (1990). Preoperative percutaneous drainage of diverticular abscesses. Am J Surg.

[B17] Gupta S, Kaushik R, Sharma R, Attri A (2005). The management of large perforations of duodenal ulcers. BMC Surg.

[B18] Gupta S, Kaushik R (2006). Peritonitis – the Eastern experience. World J Emerg Surg.

[B19] Raymond DP, May AK, Schein M, Marshall JC (2003). Acute mesenteric ischemias. Source Control.

[B20] Malangoni MA, Shumate CR, Thomas HA, Richardson JD (1990). Factors influencing the treatment of intra-abdominal abscesses. Am J Surg.

[B21] Malangoni MA (2005). Contributions to the management of intraabdominal infections. Am J Surg.

[B22] Schein M, Marshall JC (2004). Source control for surgical infections. World J Surg.

[B23] Lamme B, Boermeester MA, Belt EJ, van Till JW, Gouma DJ, Obertop H (2004). Mortality and morbidity of planned relaparotomy *versus *relaparotomy on demand for secondary peritonitis. Br J Surg.

[B24] Mazuski JE, Sawyer RG, Nathens AB, DiPiro JT, Schein M, Kudsk KA, Yowler C (2002). The Surgical Infection Society guidelines on antimicrobial therapy for intra-abdominal infections: an executive summary. Surg Infect.

[B25] Solomkin JS, Mazuski JE, Baron EJ, Sawyer RG, Nathens AB, DiPiro JT, Buchman T, Dellinger EP, Jernigan J, Gorbach S, Chow AW, Bartlett J (2003). Guidelines for the selection of anti-infective agents for the complicated intra-abdominal infection. Clin Infect Dis.

[B26] Solomon JS, Yellin AE, Rotstein OD, Christou NV, Dellinger EP, Tellado JM, Malafaia O, Fernandez A, Choe KA, Carides A, Satishchandran V, Teppler H, Protocol 017 Study Group (2003). Ertapenem versus piperacillian/tazobactam in the treatment of complicated intraabdominal infections: results of a double-blind, randomized comparative phase III trial. Ann Surg.

[B27] Malangoni MA, Song J, Herrington J, Choudri S, Pertel P (2006). Randomized control trial of moxifloxacin compared with piperacillin-tazobactam and amoxicillin-clavulanate for the treatment of complicated intra-abdominal infections. Ann Surg.

[B28] Wong PF, Gilliam AD, Kumar S, Shenfine J, O'Dair GN, Leaper DJ (2005). Antibiotic regimens for secondary peritonitis of gastrointestinal origin in adults. Cochrane Database Syst Rev.

[B29] Kollef MH (2000). Inadequate antimicrobial treatment: an important determinant of outcome for hospitalized patients. Clin Infect Dis.

[B30] Malangoni MA, Condon RE, Spiegel CA (1985). Treatment of intra-abdominal infections is appropriate with single agent or combination antibiotic therapy. Surgery.

[B31] Mosdell DM, Morris DM, Voltura A, Pitcher DE, Twiest MW, Milne RL, Miscall BG, Fry DE (1991). Antibiotic treatment for surgical peritonitis. Ann Surg.

[B32] Montravers P, Dupont H, Gauzit R, Veber B, Auboyer C, Blin P, Hennequin C, Martin C (2006). *Candida *as a risk factor for mortality in peritonitis. Crit Care Med.

[B33] Taylor E, Berjis A, Bosch T, Hoehne F, Ozaeta M (2004). The efficacy of postoperative oral antibiotics in appendicitis: A randomized prospective double-blinded study. Am Surg.

